# Multiple Chemical Sensitivity: an Italian prevalence multicentric survey

**DOI:** 10.3389/fpubh.2025.1685040

**Published:** 2025-12-05

**Authors:** Giovanni Genovese, Caterina Elisabetta Rizzo, Antonio Mistretta, Francesco Leonforte, Raffaele Squeri, Carlo Signorelli, Cristina Genovese, the Italian prevalence group

**Affiliations:** 1 Department of Biomedical Sciences and Morphological and Functional Images (BIOMORF), University of Messina, Messina, Italy; 2 Scientific Communication Unit, Istituto Superiore di Sanità, Rome, Italy; 3 Department of Medical and Surgical Sciences and Advanced Technologies “G.F. Ingrassia”, Section of Hygiene and Preventive Medicine, University of Catania, Catania, Italy; 4 Department of Integrated Hygiene, Organizational, and Service Activities (Structural Department), Health Management, University Hospital Polyclinic "G. Rodolico-San Marco", Catania, Italy; 5 School of Medicine, University Vita-Salute San Raffaele, Milan, Italy

**Keywords:** Multiple Chemical Sensitivity, environmental intolerance, QEESI, chemical exposure, hypersensitivity, epidemiology, preventive medicine

## Abstract

**Background:**

Multiple Chemical Sensitivity (MCS) is a multisystem environmental disorder triggered by exposure to chemical agents at concentrations below toxicological thresholds. Despite controversy surrounding its classification, MCS is increasingly recognized for its significant impact on health and quality of life. This study aims to evaluate the prevalence, symptomatology, and risk factors associated with MCS in the general population, using the Quick Environmental Exposure and Sensitivity Inventory (QEESI) as a diagnostic tool.

**Methods:**

A multicenter retrospective study was conducted on 494 adults. Participants completed the QEESI questionnaire, and data were analyzed for clinical correlations, laboratory findings, environmental exposures, and socio-demographic characteristics.

**Results:**

The analysis revealed that 5.7% of the population exhibited symptoms compatible with MCS. Notably, 1.4% reported changes in work or residence due to receiving an allergic or rheumatologic diagnosis. Strong associations were identified between MCS-related symptoms and exposures such as food preservatives, colorings, Wi-Fi, and chemicals the condition: Symptom severity scoring was high in 15.8% of participants, while chemical intolerance was high in 86.4%. A total of 15.5% underwent further diagnostic evaluations, with 21.4% agents. Statistical analysis highlighted correlations between MCS symptom severity and variables such as chemical intolerance, hyperosmia, hypergeusia, and dermatological reactions. Risk profiling classified 10.73% of participants as highly suggestive of MCS.

**Conclusion:**

This study confirms the presence of MCS symptoms in 5.7% of the population and highlights that a significant portion, with 10.73% being classified as “very suggestive” and 40.4% as “moderately suggestive” of the condition, may be vulnerable to chemical exposures. The results support the need for standardized diagnostic protocols and multidisciplinary approaches to management.

## 1 Introduction

Multiple Chemical Sensitivity (MCS) is as a multisystemic environmental disorder that manifests in response to chemical exposures below the tolerable threshold for the general population (1). The main implicated substances include pesticides, organic solvents, insecticides, metals, toxic gases, and molds. Symptoms affect multiple organs and systems, depending on the mechanisms through which these substances act on the human body.

The definition and classification of the syndrome remain the subject of significant scientific debate due to its complex manifestations. Nevertheless, studies have been launched in several countries, including Italy, to clarify all hypotheses about MCS. In this context, the Environmental Protection Agency (EPA) has reported that in the U.S., about one-third of individuals working in enclosed environments report a sensitivity to one or more common chemicals—a prevalence that has tripled over the past decade—leading to an estimate of approximately 55 million MCS cases.

In Italy, MCS is referred to as an Environmental Hypersensitivity or Intolerance to Xenobiotics and is categorized as a nonspecific respiratory disorder related to exposure to chemicals, gases, fumes, and vapors. The “origin” of MCS dates to 1956 when allergist Theron G. Randolph (1–3) described certain disorders as “environmental illnesses,” attributing them to an inability to adapt to various chemical compounds found in smoke, gasoline, cosmetics, and everyday objects and furnishings. In 1987, Cullen provided the first clinical definition of what is now known as MCS: “an acquired disorder characterized by recurrent symptoms involving one or more organs, triggered by demonstrable exposure to chemicals—even at concentrations far below those capable of affecting the health of the general population” (4).

It was not until 1999 that an International Consensus (5) was published, more clearly recognizing MCS and introducing a standardized tool to identify clinical characteristics in affected patients—the Environmental Exposure and Sensitivity Inventory (QEESI) (6, 7). Around the same time, the World Health Organization’s International Programme on Chemical Safety (IPCS) (8) proposed an alternative term— “Idiopathic Environmental Intolerance”—to highlight that symptoms may also be linked to non-chemical risk factors such as electromagnetic fields.

In 2005, LaCour and colleagues (9) expanded the diagnostic criteria, defining MCS as a “chronic condition lasting more than 6 months that causes a deterioration in lifestyle and physiological function; symptoms recur reproducibly, involving the nervous system and manifesting as odor hypersensitivity, with consistent involvement of the central nervous system and at least one other organ system. Symptoms are triggered even by low-level exposure to chemicals and may also respond to unrelated chemical substances. Improvement or resolution is observed upon removal from exposure.”

Toxicological studies have identified at least seven substance groups predominantly involved in MCS onset (10): organic solvents and related compounds; organophosphate and carbamate pesticides and herbicides; organochlorine insecticides; pyrethroid pesticides; hydrogen sulfide; carbon monoxide; and mercury.

Additionally, molds (11, 12) and mycotoxins (13, 14) have been implicated. Symptoms may also be triggered by exposure to bright lights, strong natural odors, noise, electromagnetic fields, and extreme temperatures (15–17).

Numerous studies have identified professional categories at higher risk of developing MCS, such as (18):

- a) industrial workers exposed to acute or chronic levels of industrial chemicals.
- b) farmers, hairdressers, and beauticians.
- c) healthcare workers, such as radiologists and anesthesiologists, engaged in hazardous tasks.
- d) teachers, students, and office workers in poorly ventilated indoor spaces (19).
- e) individuals affected by chemical accidents.
- f) residents in areas with contaminated air or aquifers (19).
- g) Gulf War veterans (20).
- h) individuals with metal or silicone prostheses (21, 22).
- i) individuals born via cesarean section (23).

Given these factors, it is evident that the general population is potentially exposed, underscoring the need for dedicated scientific studies to define the syndrome and its clinical manifestations.

The etiopathogenesis of the syndrome appears to involve the immune system, in connection with the endocrine and nervous systems, all regulated by the hypothalamic center.

Currently, several hypotheses have been proposed regarding the pathophysiological mechanisms of MCS such as Neurotoxic Hypothesis (10, 15, 24–35), Allergic and Immunologic ones (36–41), Genetic ones (42). A 1997 study (43) identified a wide range of susceptible organs and systems, showing a loss of tolerance in numerous regions, manifesting as:

- Neurological symptoms: headache, migraine, seizures, attention deficit, insomnia.
- ENT symptoms: sinusitis, nasal polyps, allergic rhinitis, recurring tinnitus.
- Cardiovascular symptoms: tachycardia, hypo/hypertension, arrhythmias, fainting.
- Respiratory symptoms: asthma, bronchospasm, tracheitis, tonsillitis.
- Gastrointestinal symptoms: irritable bowel syndrome, colitis, GERD, celiac disease, food allergies, intolerances.
- Autoimmune symptoms: carpal tunnel syndrome, arthritis, lupus, and general autoimmunity.
- Dermatological symptoms: eczema, dermatitis, rashes, urticaria, dermographism.

These generic and non-specific symptoms are often accompanied by psychological disturbances, such as anxiety, depression, bipolar disorder, and hyperarousal states—further supporting a psychosomatic component of MCS. Some current studies classify MCS under Central Nervous System Sensitization Syndromes, which also include fibromyalgia (44), chronic fatigue syndrome, and sick-building syndrome (45, 46).

Fibromyalgia (FM), chronic fatigue syndrome (CFS), Myalgia Encephalomyelitis, and Electromagnetic Hypersensitivity have been recognized as organic disorders by the World Health Organization (WHO). Specifically, FM was included in the International Classification of Diseases (ICD) in 1992 under the code M79.0, while Myalgia Encephalomyelitis was acknowledged in 1969 and later received the code ICD-11-8E49 in 2019 (47).

These conditions are believed to be caused by environmental changes and shifts in lifestyle, with significant physiological consequences in some individuals. In this framework, air pollution is implicated—alongside other factors—in the emergence and growing prevalence of various diseases.

The disorders listed above can impair memory, concentration, and attention, limit executive functions, and cause generalized psychological distress (48).

However, no clear link has emerged among self-reported MCS symptoms and widely accepted objective measures of physiological dysfunction, and no clear dose–response relationship between exposure and symptom reactions has been observed (49–53).

Recent studies have highlighted that the symptomatology of MCS is associated with objective dysfunctions in various sensory systems (54–56). At the olfactory level, metabolic alterations in cortico-subcortical areas during stimulation have been demonstrated, and it has been observed that olfactory performance can improve with specific treatments, such as the intranasal administration of hyaluronan (57, 58). The auditory system also appears to be compromised, with a peculiar lack of contralateral suppression in transient-evoked otoacoustic emissions (59). Further evidence indicates vestibular system involvement and dysregulation of the autonomic nervous system, as demonstrated by pupillographical studies (60–63). Another area of growing interest is the role of toxic metals as triggering factors. Exposure to metals has been associated with both MCS and the onset of neurogenic inflammation (64–68). In particular, the correlation between mercury released from dental amalgams and the occurrence of oral lesions has been studied, highlighting its immunotoxic and allergenic potential (69–71). Indeed, patients with MCS frequently show multiple sensitizations to metals, making screening for these elements crucial (71–74). This interaction between environmental factors and the immune system has also been implicated in autoimmune diseases (75–78).

In Italy, where Multiple Chemical Sensitivity (MCS) is not yet officially recognized as a distinct nosological entity, the lack of epidemiological data represents a major barrier for healthcare systems and for the social protection of patients (79–81). Assessing the prevalence and clinical characteristics of MCS in the Italian population is not only of scientific relevance but also has a direct societal impact: it allows estimation of the socio-economic burden of the condition, guides environmental prevention strategies, reduces costs associated with inappropriate diagnoses, and supports the development of integrated care pathways. In this perspective, the results of a multicenter Italian study can provide a concrete basis for more inclusive health policies and for institutional recognition of the disease, ultimately benefiting both patients and society at large (81, 82).

## 2 Materials and methods

A retrospective, multicenter study was designed involving the general adult population between March and October 2024.

### 2.1 Study population

The required sample size was calculated using the standard formula for estimating a population proportion:


$$\mathrm{n0}=Z^{2*}\;p(1-p)/e^{2}$$

where Z is the standard normal deviate (1.96 for a 95% confidence level), *p* is the expected prevalence (set at 0.5 to maximize variability), and e is the desired margin of error (0.05). Applying this formula yielded n0 approx. 384.16. Given the large population under study the finite population correction:


$$n=N^{*}\;\mathrm{n0}/N+n0-1$$

did not substantially alter the estimate, resulting in a minimum required sample size of 385 completed questionnaires.

### 2.2 Recruitment method

An Anonymous questionnaire was administered to participants of both sexes, who were 18 years of age and older, and who were able to understand Italian (to give informed consent and to complete the questionnaire).

Standardized questionnaires were distributed using the following techniques:

Computer-assisted personal interview (CAPI), in which an interviewer collected data during a face-to-face meeting with the interview.
Computer-assisted web interviewing (CAWI), in which the questionnaire was self-administered by the study participants and collected via email.

In the CAPI method, the interviews were conducted by medical staff (doctors/physicians) and medical residents in the Public Health and Preventive Medicine department of all participating units. This could lead to an extra-sampling error, to the interviewer’s effect.

All participants were informed about the methodology used to ensure the confidentiality of data; written informed consent was obtained in accordance with Italian privacy laws. The interviews were carried out in locations that had adequate privacy. In the CAWI method, a link was sent by the interviewers to the study participants so that they could complete the questionnaire.

### 2.3 Eligible criteria

All subjects able to complete the survey were invited to participate. Nevertheless, the main categories affected in terms of prevalence are professionally exposed workers (teachers, hairdressers, healthcare workers, industrial workers, farmers, etc.) where prolonged or repeated exposure may be a triggering factor, individuals with related conditions (common comorbidities are fibromyalgia, chronic fatigue syndrome (ME/CFS), electromagnetic hypersensitivity, multiple allergies and individuals with a genetic or familial predisposition).

The protocol of the study was approved by the Ethics Committee of the University Hospital “G. Martino” of Messina (reference number: 53-24-n°0012235), and the other Local Ethics Committee accepted it.

### 2.4 Questionnaire design

The main sections of the questionnaire were divided into:

Sociodemographic factors: age, sex, profession, place of residence, etc.…

The other part of the questionnaire consists of 50 questions divided into five main sections (83):

- Chemical exposure: evaluates reactions to common odors and substances such as cigarette smoke, paint, perfumes, cleaning products, and fuels.
- Other exposures: examines sensitivity to foods, beverages (such as caffeine and alcohol), medications, cosmetics, and materials that encounter the skin.
- Symptom severity: measures the intensity of physical and cognitive symptoms, including headaches, respiratory issues, difficulty concentrating, gastrointestinal disturbances, and mood changes.
- Impact on daily life: analyzes how sensitivities affect daily activities, social relationships, and overall quality of life.
- Masking index: identifies habitual exposures that might conceal or lessen the perception of symptoms, such as regular use of caffeine, alcohol, or medications.
- Laboratories and diagnostic test were condivided by the patients.

All data were compiled into a relational database created by the principal investigator.

### 2.5 Statistical analysis

The system included customizable data entry interfaces to allow real-time monitoring and coordination across the 11 participating centers. Data collection was continuous throughout the study period. All responses to the questionnaire were collected and summarized in Excel format.

Quantitative variables (e.g., age) were summarized using mean, median, standard deviation, range (minimum and maximum), interquartile range, and 95% confidence intervals (Table 1). Categorical variables (e.g., sex, birthplace, occupation, vaccination history) were described using absolute and relative frequencies, along with their respective 95% confidence intervals. Associations among variables were examined through contingency table analysis. Chi-square (χ^2^) tests were used for hypothesis testing, and in cases involving r × k tables, the method of partitioning degrees of freedom was applied. A significance level of *α* = 0.05 was established. Accordingly, *p*-values below 0.05 (two-tailed) were considered statistically significant. All statistical analyzes were conducted using R software.

**Table 1:** Risk criteria classification.

Suggestiveness level	Symptom score ≥40	Chemical intolerance score	Masking Index	Percentage
Very suggestive	≥40	≥40	≥4	10.73%
Very suggestive	≥40	≥40	<4	4.25%
Moderately suggestive	≥40	<40	≥4	40.40%
Not suggestive	≥40	<40	<4	23.68%
Problematic	<40	≥40	≥4	0.71%
Problematic	<40	≥40	<4	0.71%
Not suggestive	<40	<40	≥4	4.66%
Not suggestive	<40	<40	<4	6.68%

## 3 Results

The sample consisted of 494 individuals, predominantly female (M = 42.51%), with a mean age of 38.62 ± 14.12 years. Additionally, 27.5% of the sample reported engaging in physical activity, 81% consumed coffee regularly, and 58.1% were smokers.

### 3.1 Prevalence of MCS

Based on the QEESI interpretation criteria, 5.7% of participants exhibited symptoms and chemical intolerances consistent with Multiple Chemical Sensitivity (MCS) (Table 2). Moreover, 1.4% reported changing their job or residence due to environmentally related symptoms (Table 3).

**Table 2:** QEESI scoring ranges: low, medium, high.

Index	Low	Medium	High
Severe symptoms	0–19	20–39	40–100
Chemical intolerance	0–19	20–39	40–100
Other intolerances	0–11	12–24	25–100
Life impact	0–11	12–23	24–100
Masking Index	0–3	4–5	6–10

**Table 3:** Percentage distribution across scoring categories.

Index	Low	Medium	High
Severe symptoms	58.30%	23.68%	15.79%
Chemical intolerance	0.20%	12.55%	86.44%
Other intolerances	56.68%	23.28%	16.60%
Life impact	62.35%	14.78%	21.86%
Masking Index	16.40%	38.87%	25.91%

### 3.2 Clinical evaluations

Approximately 15.5% of the sample underwent laboratory testing; 14.5% received allergology consultations, with 21.4% of these resulting in a diagnosis of allergic or rheumatologic conditions. Among positive diagnostic tests, 75% revealed food and/or drug intolerances.

### 3.3 Risk classification

Analysis of QEESI-based risk criteria indicated that 10.7% of participants were “very suggestive” of MCS, and 40.4% were “moderately suggestive.” In contrast, 23.7% were classified as “not suggestive” (see Table 1).

### 3.4 Symptom severity and intolerance indices

Scoring distributions showed that:

- Severe Symptoms: 15.8% in the high range, 23.7% medium, 58.3% low.
- Chemical Intolerance: 86.4% in the high range.
- Other Intolerances: 16.6% high, 23.3% medium.
- Life Impact: 21.9% high, 14.8% medium.
- Masking Index: 25.9% high, 38.9% medium (see Tables 2, 3).

### 3.5 Disturbances and exposures

**Figure 1:**
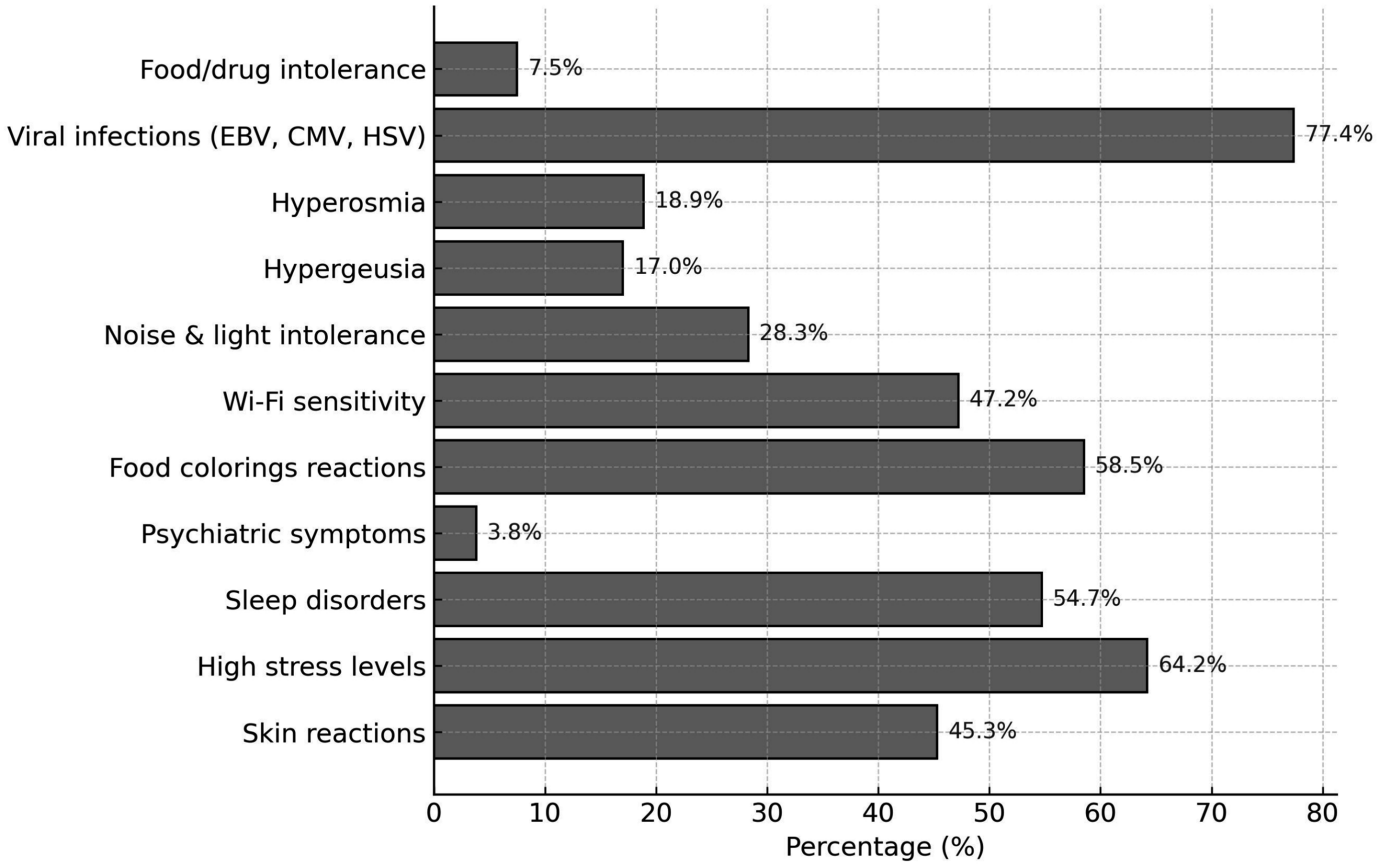
Disturbances and exposures.

Notable reported disturbances included various symptoms reported in Figure 1.

### 3.6 Significant associations

Statistical analysis identified several significant correlations (*p* < 0.05). Symptom impact was associated with skin discomfort from fabrics, Wi-Fi exposure, headaches, hyperosmia/hypergeusia, laboratory testing needs, and food/drug intolerances. Other exposures were correlated with skin irritation, Wi-Fi use, hypergeusia, and diagnostic investigations. Chemical intolerance was significantly linked to food/drug intolerance and skin discomfort. Symptom scores correlated with hypergeusia and laboratory evaluations. Masking index was associated with headaches and food/drug intolerance. Additionally, subgroup analysis (*p* < 0.001) revealed a higher prevalence of MCS-related features among individuals with implants or prostheses, farmers, anesthesiologists, industrial workers, cesarean-born individuals, and residents in polluted areas (Table 4).

**Table 4:** Sociodemographic and lifestyle characteristics of the study population (*N* = 494).

Characteristic	Category	*N* (%)
Sex	Male	209 (42.51%)
	Female	285 (57.49%)
Age (years)	Mean ± SD	38.62 ± 14.12
Physical activity	Yes	136 (27.5%)
	No	358 (72.5%)
Coffee consumption	Regular	400 (81%)
	Occasional/None	94 (19%)
Smoking status	Smoker	287 (58.1%)
	Non-smoker	207 (41.9%)

## 4 Discussion

The findings from the sample population analysis offer valuable insights into MCS and its associated conditions (84).

The presence of symptoms attributable to MCS in 5.7% of the investigated population is consistent with previous studies, which have reported a prevalence ranging from 2 to 10% across different epidemiological settings (85, 86). MCS remains a controversial condition with an as-yet unclear pathogenesis; however, the data suggest a possible link to environmental factors and genetic predisposition (87). Recent studies have highlighted the potential involvement of the central nervous system and immune system dysregulation, suggesting a complex interaction between chemical exposure and neuronal sensitization (88).

The correlation between chemical intolerances and the need for further diagnostic evaluation (15.5% of subjects) supports previous research indicating increased sensitivity to specific chemical agents in individuals with immunological and allergic disorders (89). In 21.4% of individuals presenting with MCS-related symptoms, a diagnosis of allergic or rheumatologic disease was made, supporting the hypothesis that MCS may be part of a broader clinical spectrum that includes fibromyalgia and chronic fatigue syndrome (81). Low-grade chronic inflammation, as documented in some studies, may suggest a common underlying mechanism with other conditions characterized by environmental hypersensitivity (33).

Risk criteria analysis revealed that 10.73% of subjects met the threshold for highly suggestive MCS, while 40.40% exhibited moderately suggestive symptomatology. These figures indicate a substantial portion of the population is potentially vulnerable to environmental chemical exposures, reinforcing the theory of individual susceptibility exacerbated by environmental factors (90). Additionally, early-life exposure to chemical substances may influence the risk of developing MCS in adulthood, as suggested by studies on epigenetic programming (91).

Statistically significant associations (*p* < 0.05) between chemical sensitivity and specific symptoms further strengthen prior evidence linking environmental exposure with clinical manifestations. Notably, the reported symptom impact from Wi-Fi (47.2%) and food colorings (58.5%) is of interest, although the literature remains divided on the extent to which these factors contribute to MCS (92). Some studies have proposed that exposure to electromagnetic fields may act as a trigger in genetically predisposed individuals through modulation of voltage-gated calcium channel activity (93).

The high prevalence of symptoms among workers exposed to chemicals (e.g., farmers, anesthesiologists, industrial workers) and residents in polluted areas (mean score: 47.01 ± 12.87) emphasizes the importance of environmental context in the development of MCS. These findings align with previous research indicating higher MCS prevalence among individuals in high chemical exposure settings (94). Additionally, genetic susceptibility may be influenced by polymorphisms in genes involved in detoxification and immune response, such as those in the GST and PON1 gene families (95).

Our prevalence estimate (5.7%) sits within the mid-range of international reports, which typically span ~2–10% across population-based studies and settings (80–82, 88). European surveys (e.g., Germany and Denmark) have reported comparable burdens of self-reported chemical sensitivity in community samples, whereas national studies from the United States and Australia often suggest higher population impact and greater interference with daily life (80–82, 88). Differences across countries likely reflect a combination of methodological heterogeneity—case definitions (MCS vs. IEI), screening tools and cut-offs (e.g., QEESI), sampling frames (general population vs. clinical samples), and response rates—as well as contextual factors (building ventilation standards, indoor fragrance and VOC use, occupational structures, and risk communication norms). Within Europe, where ambient pollution profiles and chemical regulations are relatively similar, the remaining heterogeneity underscores the need for harmonized measurement and pooled analyzes using standardized instruments (e.g., QEESI) and aligned thresholds. In this perspective, our multicenter Italian data add geographically balanced evidence to the European picture and may facilitate future meta-analyzes and policy benchmarking across EU member states.

## 5 Conclusion

Overall, the results point to a strong association between environmental exposures and symptomatology related to MCS and other chemical intolerances. Our findings are in line with the prevalence range reported in other national population studies (80, 81) and also in populations assessed after significant environmental events (96). This underscores the need for broader recognition of the condition and the development of specific guidelines for its diagnosis and management, given its clinical complexity. Nonetheless, further research is essential to clarify the relationship between individual predisposition and environmental factors through longitudinal studies (97–100). Moreover, the development of prevention strategies and clinical protocols is crucial to improving patient care, ideally through a multidisciplinary approach involving neurology, immunology (101–103), and environmental toxicology.

The notable finding that 5.7% of the population reported MCS-like symptoms underscores the need for broader recognition of the condition and the development of specific guidelines for its diagnosis and management, given the complexity of its clinical presentation (104–106).

Finally, the observation that 1.4% of participants had to change their jobs or residences due to illness highlights the significant socioeconomic impact of MCS (107).

In conclusion, these results underscore the urgency of establishing clear and standardized clinical guidelines for the diagnosis and management of Multiple Chemical Sensitivity (108).

## Data Availability

The original contributions presented in the study are included in the article/supplementary material, further inquiries can be directed to the corresponding authors.

## Group member of the Italian prevalence group

Aida Bianco, Department of Health Sciences, University of Catanzaro "Magna Graecia", Catanzaro, Italy; Leila Fabiani, Department of Life, Health and Environmental Sciences, University of L'Aquila, L'Aquila, Italy; Angela Di Pietro, Department of Biomedical and Dental Sciences and Morphofunctional Imaging, University of Messina, Messina, Italy; Marco Vinceti, Environmental, Genetic and Nutritional Epidemiology Research Center (CREAGEN), Department of Biomedical, Metabolic and Neural Sciences, Medical School – University of Modena and Reggio Emilia, Modena, Italy; Fabrizio Pregliasco, Department of Biomedical Sciences for Health, University of Milan, Milan, Italy; Silvana Castaldi, Department of Biomedical Sciences for Health, University of Milan, Milan, Italy; Alessandra Casuccio, Department of Health Promotion, Maternal and Infant Care, Internal Medicine and Medical Specialties (PROMISE) "G. D'Alessandro", University of Palermo, Palermo, Italy; Odone Anna, Department of Public Health, Experimental and Forensic Medicine, University of Pavia, Pavia, Italy; Caterina Rizzo, Department of Translational Research and New Technologies in Medicine and Surgery, University of Pisa, Pisa, Italy; Giovanni Boccia, Department of Medicine, Surgery and Dentistry "Scuola Medica Salernitana", University of Salerno, Baronissi Salerno, Italy; Emanuela Santoro, Department of Medicine, Surgery and Dentistry ''Scuola Medica Salernitana'', University of Salerno, Salerno, Italy; Antonio Azara, Department of Medical, Surgical and Experimental Sciences, University of Sassari, Sassari, Italy; Stefano Capolongo, Architecture, Built Environment and Construction Engineering Department, Politecnico di Milano, Milan, Italy; Roberta Siliquini, Department of Public Health, University of Torino, Italy; Paolo Villari, Department of Public Health and Infectious Diseases, Sapienza University of Rome, Rome, Italy.
